# Somatostatin receptors shape insulin and glucagon output within the pancreatic islet in mice through direct and paracrine effects

**DOI:** 10.1007/s00125-026-06769-4

**Published:** 2026-06-20

**Authors:** Ryan G. Hart, Jordan J. Lee, Karen Zhai, Sharlene Lee, Rashita Chauhan, Aidean Hosseini, Austin D. Nguyen, Mark O. Huising

**Affiliations:** 1 Department of Neurobiology, Physiology and Behavior, University of California Davis, Davis, CA USA; 2 Department of Physiology and Membrane Biology, University of California Davis, Davis, CA USA

**Keywords:** Alpha cell, Beta cell, Calcium, Cyclic AMP, Delta cell, GCaMP6, Islet biology, Live imaging, Second messenger, Somatostatin, SST

## Abstract

**Aims/hypothesis:**

Pancreatic delta cells secrete somatostatin (SST), which can inhibit both alpha cells and beta cells of the pancreatic islet. By controlling insulin and glucagon release, delta cells play an important role in maintaining nutrient homeostasis. However, the mechanism by which a single inhibitory hormone inhibits both alpha cells and beta cells, which are often considered as functional antagonists in the counterregulatory control of blood glucose, has been a physiological riddle. Here, we solve this riddle through assessment of the contributions of alpha cell-specific and beta cell-specific SST receptors to cell-intrinsic behaviours and hormone release.

**Methods:**

Islets from mice constitutively expressing fluorescent sensors reporting on cyclic AMP and Ca^2+^ in both alpha and beta cells were imaged using stimuli to mimic the postprandial state of a meal consisting of glucose and amino acids. This approach was coupled with cell-specific SST receptor antagonists to identify how SST inhibits alpha and beta cell hormone output through modulation of cAMP and Ca^2+^ second messengers and paracrine interactions.

**Results:**

Our results support and extend prior observation that SST receptor 2 (SSTR2) is the only SST receptor expressed by mouse alpha cells, while SST receptor 3 (SSTR3) is the only receptor expressed by beta cells. Interestingly, SSTR2 and SSTR3 regulate downstream cAMP and Ca^2+^ signalling cascades differently within alpha and beta cells of intact islets. Stimulation of SST receptors robustly inhibits cyclic AMP in alpha cells and beta cells. In contrast, stimulation of SSTR2 inhibits alpha cell Ca^2+^ with significantly greater potency compared with inhibition of beta cell Ca^2+^ via SSTR3. Despite the absence of SSTR2 on beta cells, blocking alpha cell SSTR2 during nutrient stimulation resulted in a significant increase in insulin release downstream of local release of glucagon.

**Conclusions/interpretation:**

Our observations address the physiological riddle of the delta cell’s role during the postprandial phase where we demonstrate that SST primarily inhibits alpha cell cAMP and Ca^2+^ via SSTR2, preventing glucagon release. Blocking SSTR2 results in an increase in locally released glucagon, which coupled with muted ability for SSTR3 to inhibit beta cell calcium under strong nutrient stimulation, results in potentiation of glucose-stimulated insulin secretion from the beta cell. We conclude that the role of delta cells under nutrient stimulation is to modulate the volume of insulin release by tuning the strength of intra-islet paracrine potentiation of insulin secretion by glucagon, mediated via beta cell glucagon-like peptide-1 receptors.

**Code availability:**

All code used for analyses and data processing are available on GitHub (https://github.com/Huising-Lab/Hart-et-al.-Diabetologia-2026).

**Graphical Abstract:**

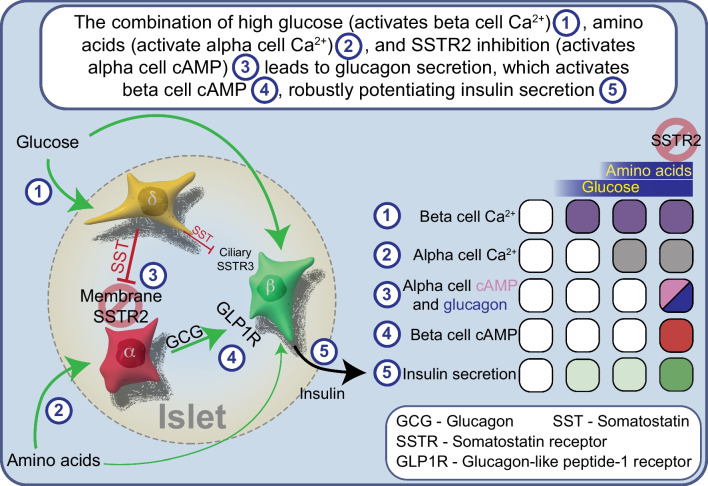

**Supplementary Information:**

The online version contains peer-reviewed but unedited supplementary material available at 10.1007/s00125-026-06769-4.


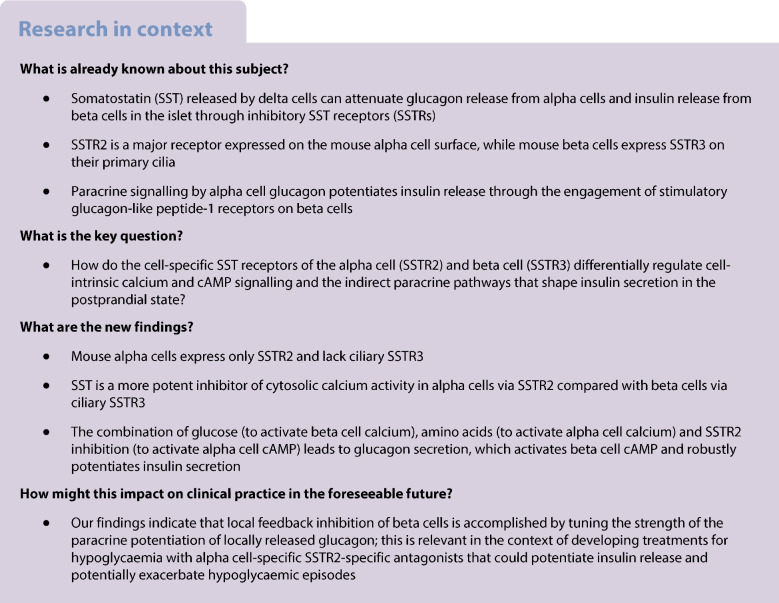


## Introduction

A tightly regulated network of pancreatic islet cells is responsible for maintaining nutrient homeostasis [1]. Two of these cell types, alpha cells and beta cells, achieve this by balancing the secretion of glucagon and insulin, respectively [2]. Glucagon is an important counterregulatory hormone that elevates blood glucose under hypoglycaemic conditions by stimulating hepatic glucose production [3]. Insulin promotes the uptake and storage of nutrients to lower circulating blood glucose [4]. The delta cell is the third main islet endocrine cell type, secreting the hormone somatostatin (SST) [5], which was recognised as a local inhibitor of alpha and beta cell hormone secretion shortly after its discovery [6]. Recently, we demonstrated how feedback inhibition provided by delta cells is coordinated with beta cells [7] and sets the homeostatic set point for blood glucose between meals [8]. However, how a single inhibitory hormone, SST, simultaneously inhibits both alpha and beta cells, has not been satisfactorily explained. Under a counterregulatory model where glucagon and insulin are released at low and high glucose concentrations, respectively, it would not make physiological sense to contemporaneously inhibit secretion of both hormones. Several recent papers demonstrate that glucagon during the fed state serves as a local amplifier of nutrient-stimulated insulin secretion via the activation of beta cell glucagon-like peptide-1 receptor (GLP1R) [9–13], affirming long-standing observations that glucagon stimulates insulin release [14]. These observations reframe alpha cells as cooperative partners with beta cells during the postprandial phase [9, 10]. This simplifies the role of postprandial delta cell SST, as either direct inhibition of beta cells or indirect inhibition by the local release of alpha cell-derived glucagon would converge to suppress insulin secretion.

SST signals through a family of five different inhibitory G-protein-coupled (Gαi GPCR) SST receptors (SSTRs), SSTR1–5 [15]. Comprehensive transcriptomes of FACS-purified alpha cells and beta cells have established *Sstr3* mRNA as the only *Sstr* mRNA present in mouse beta cells, whereas mouse alpha cells express *Sstr2* and *Sstr3* [16–19]. All SSTRs localise to the cell membrane, except for SSTR3, which is expressed on the primary cilia [20]. Whether the differences in localisation of SSTR2 and SSTR3 affect downstream signalling and their potency to inhibit islet hormone release has not been determined. Activation of the Gαi subunit inhibits adenylyl cyclase to reduce the production of cAMP [21]. SST can inhibit cytosolic calcium (Ca^2+^) via β/γ trimeric protein-mediated activation of inward rectifying potassium (GIRK) channels [22] or sodium–potassium ATPase transporters in primary beta cells [23].

Several gaps in our understanding of SST-mediated feedback remain, including whether SST inhibits insulin secretion directly through beta cell SSTR3 or indirectly by attenuating paracrine stimulation of insulin release by glucagon from alpha cells. We also do not know the relative importance of SST inhibition of intracellular Ca^2+^ or cAMP in alpha cells and beta cells. To close these knowledge gaps, we leveraged mice expressing genetically encoded sensors reporting on both Ca^2+^ and cAMP in all islet cells to quantify the inhibitory effects of SST on primary alpha and beta cells.

## Methods

### Mouse strains

C57BL/6NHsd mice were group housed (up to four mice per cage; Techniplast, corncob bedding with crinklet) in a specific pathogen-free facility on a 12 h light–dark cycle, at 20–26°C and 30–70% humidity with ad libitum access to water and standard rodent chow. Constitutive GCaMP6s Ca^2+^ indicator mice were generated by crossing heterozygous lox-stop-lox GCaMP6s mice (B6;129S6-*Gt(ROSA)26Sor*^tm96(CAG-GCaMP6s)Hze^/J, https://www.jax.org/strain/024106) [24] with a heterozygous β-actin-Cre mouse (B6N.FVB-Tmem1636^TG(ACTB-Cre)2 Mrt^/CjDswJ, https://www.jax.org/strain/019099) [25], resulting in germline removal of the stop cassette. Constitutive cAMP sensor mice were generated similarly using heterozygous lox-stop-lox CAMPER mice (Gt(ROSA)26Sor^tm1(CAG-ECFP*/Rapgef3/Venus*)Kama^/J, https://www.jax.org/strain/032205) [26]. Islet-cell-specific membrane tdTomato lox-stop-lox membrane GFP (mT/mG) mice were generated by crossing a heterozygous mT/mG mouse (B6.129(Cg)-Gt(ROSA)26Sor ^tm4(ACTB-tdTomato,-EGFP)Luo^/J, https://www.jax.org/strain/007676) [27] with a heterozygous mouse expressing alpha cell-specific (GCG-CreEr *Gc*^*gem1(cre/ERT2)*^*,* https://www.mmrrc.org/catalog/sds.php?mmrrc_id=42277) [28], beta cell-specific (Tg(Ucn3-Cre)KF43Gsat/Mmucd, https://www.mmrrc.org/catalog/sds.php?mmrrc_id=32078) [29] or delta cell-specific (SST-Cre (*Sst*^*t*m2.1(Cre)Zjh^/J, https://www.jax.org/strain/013044) [30] Cre drivers. Imaging experiments were performed with mice between 3 months and 6 months of age of both sexes. All mouse experiments were approved by the UC Davis Institutional Animals Care and Use Committee and performed in compliance with the Animal Welfare Act and the Institute for Laboratory Animal Research Guide to the Care and Use of Laboratory Animals.

### Islet isolation and dissociation

Islets were isolated as described [7, 8] and cultured in RPMI (Gibco, 11879-020) supplemented with FBS (10% wt/vol.) (Gibco, A5256701), penicillin/streptomycin (50,000 U total) (Gibco, 15140122) and 5.5 mmol/l glucose (complete) for 16–24 h. For dissociation, islets were handpicked into 15 ml conical vials, gravity sedimented and triturated in 500 μl 0.25% trypsin (Gibco, 25200056), then suspended in RPMI. Pelleted (5 min at 500 *g*) and dissociated cells were seeded at 30,000 cells/imaging chamber.

### Static hormone secretion assays

After overnight culture (RPMI, 5.5 mmol/l glucose), pooled islets from age-matched C57BL/6NHsd mice (Envigo) were picked twice into Krebs Ringer Buffer (20 mmol/l HEPES pH 7.4, 1.2 mmol/l KH_2_PO_4_, 25 mmol/l NaHCO_3_, 130 mmol/l NaCl, 5 mmol/l KCl, 1.2 mmol/l MgCl_2_, 1.2 mmol/l CaCl_2_) containing 0.1% BSA and 5.5 mmol/l glucose, then incubated at 37°C for 1 h. Glucagon and insulin secretion were measured in parallel from 20 pooled islets in 24-well plates with at least five replicates per condition by Lumit luminescence assay kit (Promega). Samples with luminescence above or below the range of the standard curve were omitted.

### Live imaging of second messenger levels

Intact islets or dissociated islet cells were seeded into microperfusion chambers bonded to a 35 mm glass-bottom dish (Mattek, P35G-1.5-14-C) and allowed to adhere for 24–48 h before imaging [8]. Continuous perfusion of KRB was maintained at 200 μl/min [7, 8]. The Ca^2+^ and cAMP responses of islets over time were imaged using a Nikon Eclipse Ti2 using a 60× lens (N.A. 1.4) with oil for intact islets, or a 20× non-immersion objective (N.A. 0.75) for dissociated cultures. Somatostatin-14 (SST-14) (Bachem, 4033009) was perfused at 100 nmol/l to activate SSTRs during imaging. Stimulation with 100 nmol/l adrenaline (epinephrine, Sigma, e4642) at the conclusion of each trace identified alpha cells (robustly stimulated via β_1_ adrenergic receptors) and beta cells (potently inhibited via α_2_ adrenergic receptors). Intact imaging experiments with isolated islets from at least three mice were combined.

### Immunofluorescence

Pancreases were cryosectioned and immunostained as described previously [7, 8, 31]. Whole-mount staining of intact islets following live imaging was carried out as described with validated primary antibodies (see electronic supplementary material [ESM] Table 1) [7, 8, 32] on a Nikon Eclipse Ti using a 60× lens with oil [7, 8, 32].

### Analysis and quantification of SSTR3-positive cilia across islet cell lineages

Analysis of membrane GFP or membrane tdTomato colocalisation with SSTR3 was performed in ImageJ [33] by marking each SSTR3-positive cilium and comparing against both the GFP and tdTomato acquisitions of the same field. Cilia with no overlapping membrane signal were excluded. File names were scrambled to ensure rigour and prevent bias during analysis.

### Analysis of live imaging results

Multi-image time series of islet behaviour were acquired as described [7, 8, 32] and analysed using Python v3.8 (code available at https://github.com/Huising-Lab/Hart-et-al.-Diabetologia-2026). We used generative artificial intelligence to efficiently generate and edit our code for analysis; all outputs were inspected before incorporating into datasets. To account for lateral drift when imaging, an affine stabilisation with a temporal window size of ten frames was applied to every time series. A median projection of each islet was used to segment individual cells using CellPose [34]. Cells were segmented into regions of interest (ROI), then eroded by 10% to limit overlap between individual cells within the islet. Cells that drifted out of the focal plane during the duration of the recording were excluded from analysis for technical reasons. Mean intensity values were used to quantify ROI-specific behaviours normalised per ROI. Cell identity was assigned through a first filter using a *t* test on the adrenaline response against the 5 min preceding baseline. Signal bleed-through from neighbouring beta cells into alpha cell Ca^2+^ ROIs was removed by mapping nearest neighbour beta cell ROIs (ESM Fig. 1, ESM Video 1). The constitutive cAMPER Forster resonance energy transfer (FRET) SH74 cAMP biosensor [26] leverages photon exchange between the donor (CFP) and acceptor (YFP). FRET ratios for individual cells were calculated using the formula (CFP^ROI^)/(YFP^ROI^) with intensity data from each channel collected independently, followed by normalisation of each individual cell. Identity of each cell was confirmed by post hoc immunofluorescence of glucagon and insulin.

### Adenovirus generation and transduction

Recombinant jRGECO1a [35] (Addgene 100852) adenoviral particles were generated using the AdEasy system [36]. Adenoviral particles were delivered at a multiplicity of infection of 50 for a period of 12–16 h to dissociated islet cells.

### Statistical analysis

Comparisons within the same time series of the same cell were performed using a paired Student’s *t* test (*p*<0.05). Comparisons between cell populations were tested by Student’s *t* test (*p*<0.05). Comparisons between groups of treatments were tested by one-way ANOVA (*p*<0.05). Analyses were performed in GraphPad Prism (Version 10) with raw data processed using Python (v3.8 https://www.python.org/) operated from a Jupyter notebook (https://jupyter.org/).

## Results

### Alpha cells express SSTR2 and beta cells express SSTR3 in mice

**Fig. 1:**
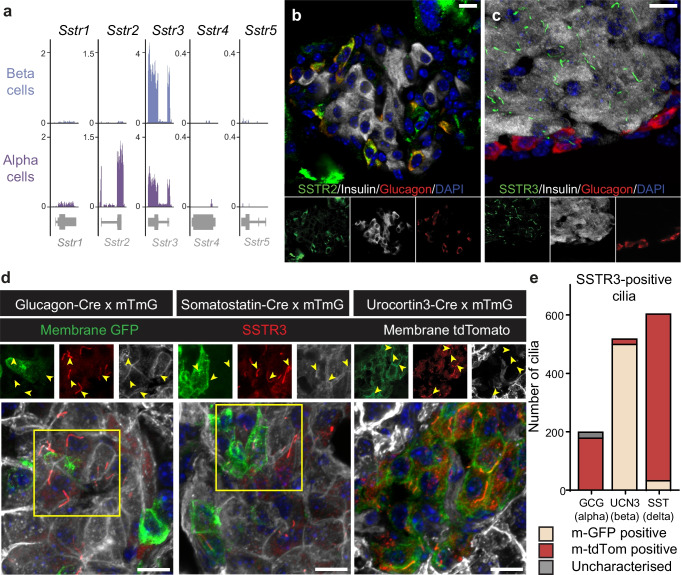
Alpha cells express SSTR2 and beta cells express SSTR3 in mice. (**a**) Bulk alpha and beta cell RNA-seq results identified *Sstr3* message present on both alpha and beta cells and *Sstr2* message present only in the alpha cells, with *Sstr1*, *Sstr4* and *Sstr5* message absent from both cell types. (**b**, **c**) Immunostaining of SSTR2 and SSTR3 protein expression identified SSTR2 on exclusively alpha cells (**b**), while SSTR3 was expressed on primary cilia of islet cells (**c**) (scale bar, 10 µm). (**d**) SSTR3-positive cilia cell identity was confirmed against colocalisation with a membrane GFP signal delivered by cell-specific Cre driver. Yellow arrows indicate position of cilia observed within either the entire field of view or within the yellow boxed region. The vast majority of cilia that overlapped with the GFP cell identity marker were those from the beta cell (scale bar 10 µm). The membrane tdTomato is pseudo-coloured white to accentuate the overlap between SSTR3 and the membrane GFP. (**e**) Quantification of the overlapping SSTR3 and membrane GFP or membrane tdTomato confirmed that the majority of SSTR3-positive cilia were localised to the beta cell of the islet

Previously published datasets identified the specific *Sstr* mRNA profiles for FACS-purified alpha cells and beta cells of the islet (Fig. 1a) [16]. SSTR2 colocalised with glucagon-positive alpha cells (Fig. 1b). SSTR3 expression was limited to the primary cilia (Fig. 1c). Cell identity of SSTR3-positive cilia was determined by labelling alpha, beta or delta cell cilia with Cre-driven membrane GFP while lineage-negative cilia were labelled with membrane tdTomato (Fig. 1d). Quantification of the colocalisation between these signals confirmed that most beta cell cilia express SSTR3; however, no SSTR3 protein was detected on alpha cells and very little on delta cells (Fig. 1e), despite detectable *Sstr3*.

### SST signalling attenuates Ca^2+^ in alpha cells more than in beta cells

**Fig. 2:**
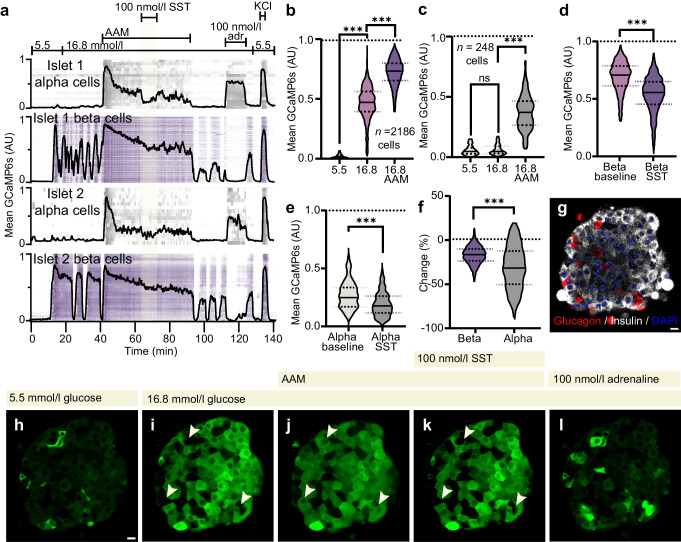
SST signalling attenuates Ca^2+^ in alpha cells more than in beta cells. Thirty one β-actin-Cre × GCaMP6s islets were analysed across four separate mice, resulting in analysis of 248 alpha cells and 2186 beta cells. (**a**) Islets were stimulated using both 16.8 mmol/l glucose and an AAM (2 mmol/l of l-glutamine, l-alanine and l-arginine each) to drive Ca^2+^ transients. Two representative islet responses are shown. (**b**, **c**) Treatment with high glucose significantly increased Ca^2+^ in beta cells (**b**) but not in alpha cells (**c**). Treatment with high glucose and the AAM significantly increased Ca^2+^ in both alpha cells and beta cells (one-way ANOVA ***p<0.001). (**d**, **e**) A 10 min treatment with 100 nmol/l SST significantly reduced both beta cell (**d**) and alpha cell (**e**) Ca^2+^ intensity compared with the 10 min baseline intensity immediately preceding treatment (paired Student’s *t* test, ***p<0.001). (**f**) Comparison of the percent drop in GCaMP6s intensity revealed a significantly larger effect of SST in inhibition of alpha cell Ca^2+^ (Student’s *t* test, ***p<0.001). (**g**) Representative immunofluorescence of imaged islet revealed alpha and beta cell fate as stained by glucagon and insulin (scale bar, 10 µm). (**h**–**l**) Still images of representative islets highlighted selectiveness of metabolic stimuli and cell behaviour in response to experimental stimuli (scale bar, 10 µm). White arrows indicate alpha cells identified by both adrenaline response and immunofluorescence. Violin plots (**b**–**f**) present the median as a solid line, with the 25th and 75th percentiles marked as dashed lines. adr, adrenaline; AU, arbitrary units; ns, not significant


We next compared the ability of SST to inhibit simultaneous Ca^2+^ responses in alpha and beta cells. We stimulated islets from constitutive GCaMP6s reporter mice with 16.8 mmol/l glucose and added an amino acid mixture (AAM) (2 mmol/l of l-glutamine, l-alanine and l-arginine each) to reflect the postprandial presence of carbohydrates and protein. This tonically activated Ca^2+^ in both alpha and beta cells (Fig. 2a). We observed a significant increase in beta cell Ca^2+^ in response to both high glucose and subsequent addition of amino acids (Fig. 2b). In alpha cells, Ca^2+^ increased in response to amino acids but not high glucose alone (Fig. 2c). Subsequent application of SST-14 (100 nmol/l) caused a significant drop in Ca^2+^ signal in both alpha cells and beta cells (Fig. 2d, e) that was greater in alpha cells than in beta cells (Fig. 2f). Cell identity was confirmed by the response of each individual cell to adrenaline, and by comparing post hoc immunofluorescence for insulin and glucagon (Fig. 2g) with still images from this experiment (Fig. 2h–i and ESM Video 2).

ESM Video 2 Mouse islet expressing GCaMP6s in alpha, beta and delta cells. Audio corresponds to average intensity of beta cells (lower pitch) and alpha cells (higher pitch) Corresponds to Figure 2. (MP4 6551 KB)

### Dissociation of intact islets increases beta cell sensitivity to SST

**Fig. 3:**
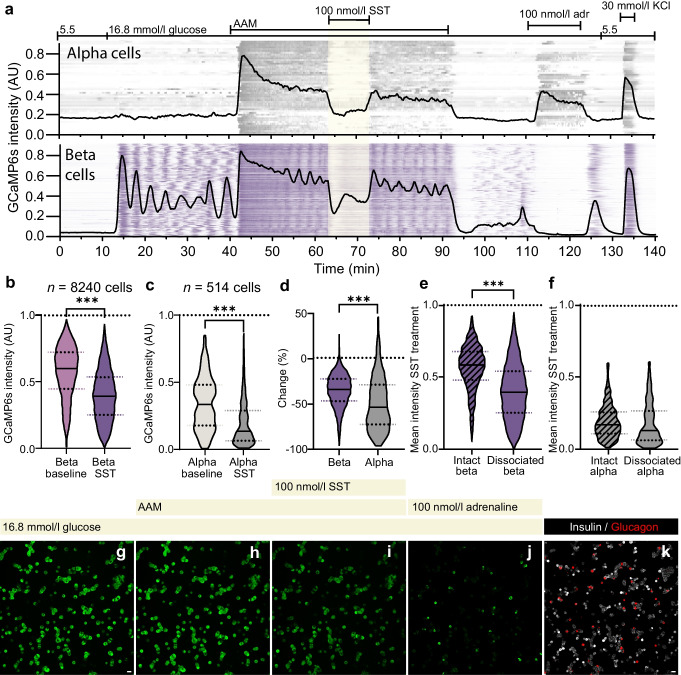
Dissociation of intact islets increases beta cell sensitivity to SST. Four separate dissociated cultures from pooled β-actin-Cre × GCaMP6s mice were generated (10 mice in total). A total of 8240 beta cells and 514 alpha cells were assessed across imaging experiments. (**a**) Representative Ca^2+^ intensity in response to stimuli identical to the intact islets is presented across one imaging experiment, with all cells combined in one graph and overlaid intensity plot. (**b**, **c**) Treatment with 100 nmol/l SST resulted in a significant drop in GCaMP6s fluorescence in both beta cells (**b**) and alpha cells (**c**) when comparing the SST treatment period with the 10 min baseline in the glucose + AAM treatment immediately preceding (paired Student’s *t* test, ***p<0.001). (**d**) Comparison of the percent drop in GCaMP6s intensity revealed a significantly larger effect of SST in inhibition of alpha cell Ca^2+^ (Student’s *t* test, ***p<0.001). (**e**, **f**) Comparing the mean normalised value during SST treatment in intact beta cells and intact alpha cells against dissociated cell populations revealed that beta cells were more sensitive to 100 nmol/l SST treatment when dissociated (**d**) while alpha cell inhibition was comparable between those in the intact or dissociated conformation (**e**) (Student’s *t* test, ***p<0.001). (**g**–**j**) Still images illustrate cell behaviour in response to metabolic stimuli and treatments (scale bar, 20 µm). (**j**, **k**) Immunofluorescence of glucagon and insulin coupled with adrenaline response identified alpha and beta cells (scale bar, 20 µm). Violin plots (**b**–**f**) present the median as a solid line, with the 25th and 75th percentiles marked as dashed lines. adr, adrenaline; AU, arbitrary units

Gap junction coupling between beta cells may buffer SST-mediated Ca^2^⁺ inhibition, while ciliary localisation of SSTR3 may further compartmentalise downstream cytosolic Ca^2^⁺ signalling cell-autonomously [37, 38]. To differentiate between these scenarios, we dissociated constitutive GCaMP6s islets and imaged them across several thousand beta cells (*n*=8240) and several hundred alpha cells (*n*=514). Dissociation physically removes gap junctions, while retaining SSTR3 expression on primary cilia (ESM Fig. 2). The overall response of alpha and beta cells to SST (Fig. 3a) was comparable with their responses in intact islets (Fig. 2a). SST-14 significantly inhibited Ca^2+^ in individual alpha cells and beta cells, with the response being more robust in alpha cells (Fig. 3b–d). Normalised Ca^2+^ was more robustly inhibited by SST in dissociated beta cells (Fig. 3b) than in beta cells in intact islets (Figs 2a, 3e), while SST inhibited Ca^2+^ at a similar magnitude in dissociated and intact alpha cells. (Figs 2c, 3f) Still images of dissociated islet cell behaviour coupled with immunostaining confirmed cell identity (Fig. 3g–j and ESM Video 3).

### SST robustly attenuates cAMP in beta cells and alpha cells

**Fig. 4:**
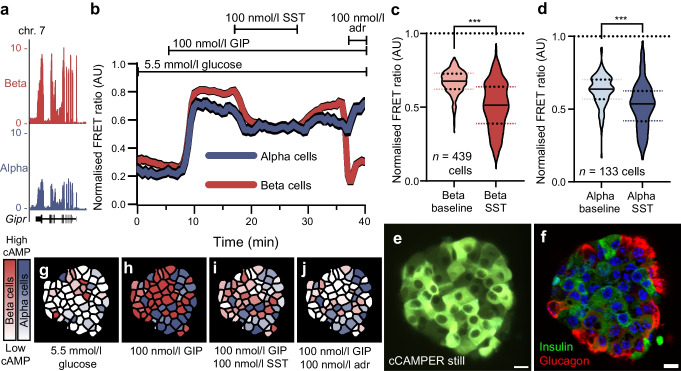
Somatostatin robustly attenuates cAMP in beta cells and alpha cells. Nine β-actin-Cre × CAMPER islets were imaged from three mice on separate days. Across all imaging experiments, 439 beta cells and 133 alpha cells were imaged. (**a**) Mouse alpha cell and beta cell transcriptomes identified meaningful *Gipr* expression in both cell types. (**b**) Example data from one islet are presented during treatment in basal glucose (5.5 mmol/l) with 100 nmol/l GIP stimulating cAMP production in alpha cells and beta cells, and 100 nmol/l SST applied to track inhibition. (**c**, **d**) Treatment with 100 nmol/l SST resulted in a significant decrease in cAMP in both alpha cells and beta cells (paired Student’s *t* test, ***p<0.001). (**e**, **f**) Representative still of cAMP expressing islet and corresponding immunofluorescence confirm cell identity and islet structure (scale bar, 10 µm). (**g**–**j**) The islet response to GIP and SST is presented as pseudo-coloured cell masks to track both cell types and changes over time. Violin plots (**c**, **d**) present the median as a solid line, with the 25th and 75th percentiles marked as dashed lines. adr, adrenaline; AU, arbitrary units

Having determined that SST inhibited Ca^2+^ generated by nutrient stimulation with greater potency in alpha cells than in beta cells (Fig. 2), we next determined the ability of SST to inhibit cAMP in both cell types. We stimulated islets from constitutive CAMPER (cCAMPER) reporter mice with gastric inhibitory polypeptide (GIP) to activate a robust cAMP response in alpha cells and beta cells (Fig. 4a) [16, 39]. This enabled quantification of cAMP inhibition upon addition of SST-14 (100 nmol/l) in alpha cells and beta cells (Fig. 4b and ESM Video 4). SST inhibited cAMP levels to a similar extent in alpha cells and beta cells (Fig. 4c, d), the identity of which was confirmed by a combination of adrenaline responses (Fig. 4b) and post hoc immunofluorescence coupled with live imaging results (Fig. 4e–j).

### Selective SSTR inhibition relieves Ca^2+^ attenuation in alpha and beta cells

**Fig. 5:**
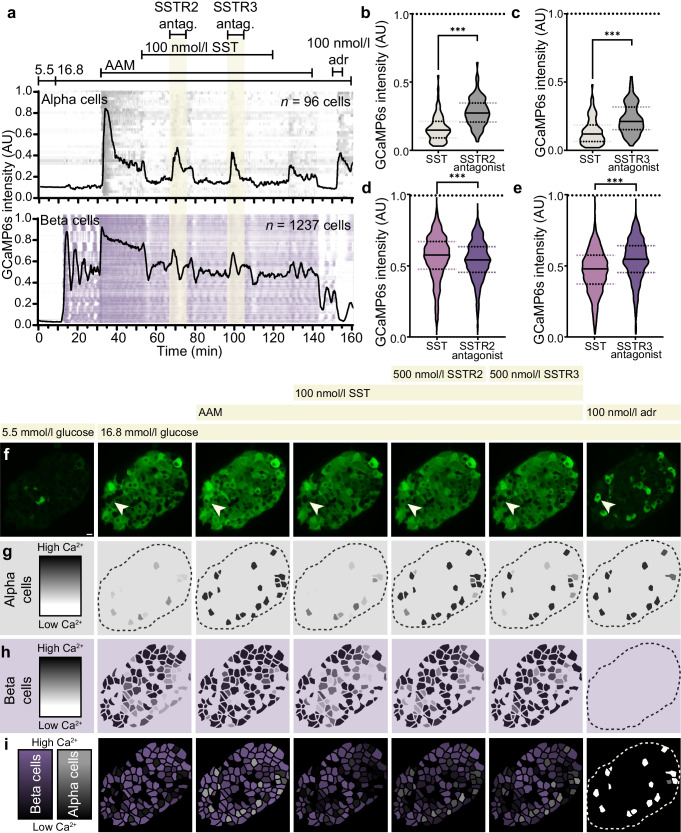
Selective SSTR inhibition relieves Ca^2+^ attenuation in alpha cells and beta cells. Sixteen β-actin-Cre × GCaMP6s islets from three separate mice were imaged, resulting in data acquired from 96 alpha cells and 1237 beta cells. Islets were subjected to the same series of metabolic stimuli as in Fig. 2 but with a prolonged 100 nmol/l SST treatment to establish an inhibitory baseline. The SST-mediated baseline was used to introduce 500 nmol/l of SSTR2 (406-028-15) and SSTR3 (SST-ODN-8) antagonists. (**a**) Line graphs and intensity plots represent the average intensity of all cells acquired and each individual cell. (**b**, **c**) Both the SSTR2 (**b**) and the SSTR3 antagonist (**c**) yielded significant relief of SST-mediated Ca^2+^ inhibition in alpha cells (paired Student’s *t* test, ***p<0.001). (**d**, **e**) In beta cells, SSTR2 antagonism (**d**) resulted in a significant decrease in Ca^2+^ from the already suppressed SST baseline and treatment with the SSTR3 antagonist (**e**) significantly relieved SST attenuation of Ca^2+^ (paired Student’s *t* test, ***p<0.001). (**f**) Still images highlight a representative islet response to stimuli and peptides over the course of an imaging experiment (scale bar, 10 µm). The white arrow highlights a representative alpha cell across numerous treatments. (**g**, **h**) Cell-specific ROIs were mapped to alpha and beta cell-specific ROIs to highlight cell behaviour across treatments. (**i**) Mapped ROIs were merged to ease the visualisation of separate cell behaviours. Violin plots (**b**–**e**) present the median as a solid line, with the 25th and 75th percentiles marked as dashed lines. adr, adrenaline; antag., antagonist; AU, arbitrary units


To validate the specificity of the inhibitory effects of SST on alpha cell Ca^2+^ (SSTR2) and beta cell Ca^2+^ (SSTR3), islets from GCaMP6s reporter mice were stimulated as before (Fig. 2), followed by SST-14 (100 nmol/l) to establish an ‘inhibitory baseline’ (Fig. 5a). Subsequent application of the selective SSTR2 antagonist 406-028-15 (500 nmol/l) [40] and the SSTR3-selective antagonist SST-ODN-8 (500 nmol/l) [41] both significantly relieved alpha cell Ca^2+^ inhibition (Fig. 5b, c). Antagonism of SSTR2 also initially relieved beta cell Ca^2+^ followed by a sharp decrease in Ca^2+^ (Fig. 5a, d). The SSTR3 antagonist relieved SST-mediated Ca^2+^ inhibition in beta cells (Fig. 5e). Visualisation of Ca^2+^ behaviour in response to select stimulations demonstrated how much more robust the effect of SST inhibition is on alpha cells compared with beta cells (Fig. 5f–i and ESM Video 5).

ESM Video 5 Mouse islet expressing GCaMP6s in alpha, beta and delta cells. Audio corresponds to average intensity of beta cells (lower pitch) and alpha cells (higher pitch) Corresponds to Figure 5. (MP4 5915 KB)

### Selective SSTR inhibition relieves cAMP attenuation in alpha and beta cells

**Fig. 6:**
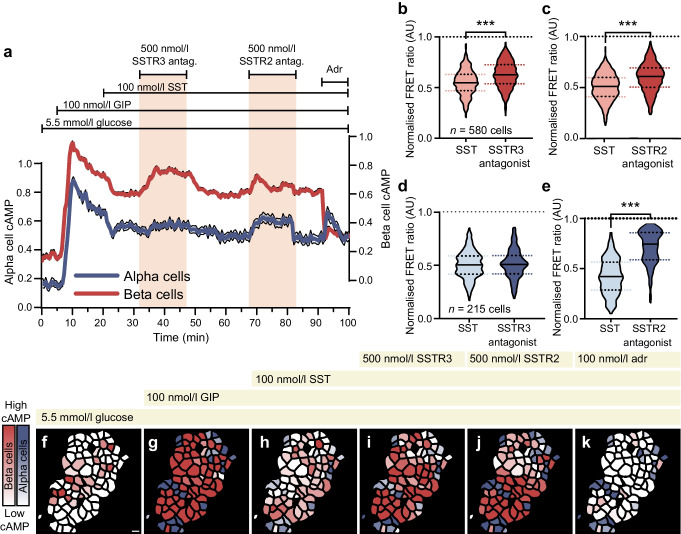
Selective SSTR inhibition relieves cAMP attenuation in alpha cells and beta cells. Fifteen β-actin-Cre × CAMPER islets were imaged from four separate mice with a total cell count of 215 alpha cells and 580 beta cells. (**a**) A tonic SST inhibition baseline was established against the background of basal glucose (5.5 mmol/l) and sustained GIP (100 nmol/l). (**b**, **c**) Against this background, beta cell cAMP was significantly increased in response to SSTR3 (**b**) and SSTR2 antagonism (**c**) (paired Student’s *t* test, ***p<0.001). (**d**, **e**) In alpha cells, SSTR3 antagonism (**d**) had no effect on cAMP but SSTR2 antagonism (**e**) significantly elevated cAMP activity (paired Student’s *t* test, ***p<0.001). (**f**–**k**) Pseudo-coloured images of an islet treated with the stimuli in (**a**) are shown to highlight changes in alpha and beta cell cAMP (scale bar, 10 µm). Violin plots (**b**–**e**) present the median as a solid line, with the 25th and 75th percentiles marked as dashed lines. adr, adrenaline; antag., antagonist; AU, arbitrary units


Next, we quantified the effects of selective SSTR2 and SSTR3 antagonists on alpha and beta cell cAMP responses using islets from cCAMPER mice (Fig. 6a). As expected, blocking SSTR3 relieved the inhibition of cAMP by SST on beta cells (Fig. 6b). However, selective blockade of SSTR2 resulted in a significant reprieve from SST-mediated inhibition of cAMP in beta cells (which do not express SSTR2) (Fig. 6c). In alpha cells, SSTR3 antagonism had no effect on SST-mediated inhibition of cAMP (Fig. 6d), while the SSTR2 antagonist significantly relieved SST-mediated inhibition of alpha cell cAMP (Fig. 6e), in line with expression of SSTR2 (Fig. 1b). Pseudo-coloured islet maps of cell-specific ROIs highlight the response of each stimulation, with adrenaline distinguishing alpha and beta cell cAMP responses (Fig. 6f–k and ESM Video 6).

ESM Video 6 Alpha (blue) and beta (red) cell ROI from a mouse islet expressing a cAMP sensor H74 in all cells (cCAMPER mouse). Corresponds to Figure 6. (MP4 3484 KB)

### Islet dissociation removes SSTR2-mediated relief of beta cell cAMP

**Fig. 7:**
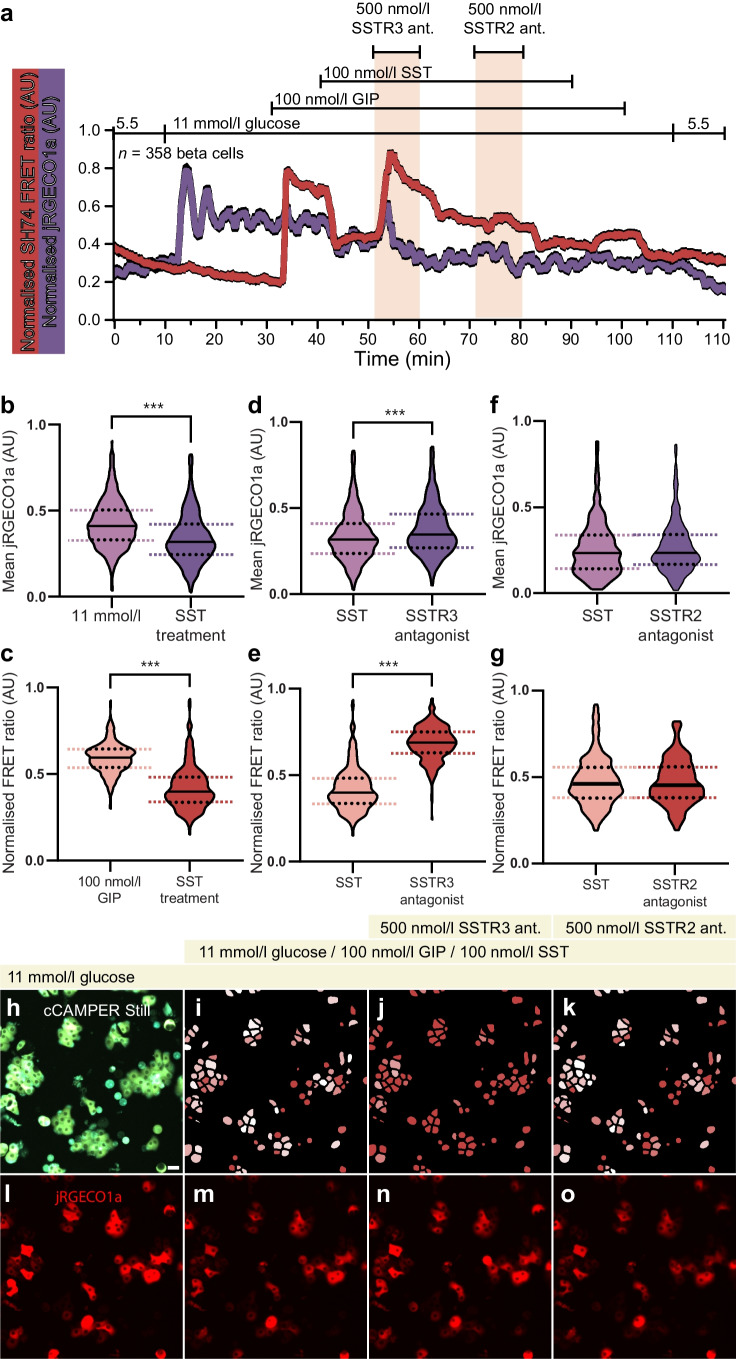
Islet dissociation removes SSTR2-mediated relief of beta cell cAMP. To assess beta cell Ca^2+^ and cAMP simultaneously, a beta cell-specific Cre driver was crossed with the CAMPER mouse and islets were transduced with the red Ca^2+^ sensor jRGECO1a. This experiment was performed with pooled islets from two mice, dissociated and seeded together. This yielded a total of 358 beta cells. (**a**) Islets were dissociated and subjected to the same strategy of stimulating the cells and establishing a tonic SST inhibitory baseline to test SSTR2 and SSTR3 antagonist effects. (**b**–**e**) In dissociated beta cells, Ca^2+^ and cAMP were significantly inhibited by 100 nmol/l SST (**b**, **c**), with inhibition of both relieved by the SSTR3 antagonist (**d**, **e**) (paired Students’ *t* test, ***p<0.001). (**f**, **g**) Addition of the SSTR2 antagonist resulted in no significant relief of SST inhibition of either cAMP or Ca^2+^. (**h**–**o**) Representative dissociated cultures expressing the genetically knocked in CAMPER sensor and virally delivered jRGECO1a sensor are displayed (scale bar, 20 µm). Violin plots (**b**–**g**) present the median as a solid line, with the 25th and 75th percentiles marked as dashed lines. Ant., antagonist; AU, arbitrary units

To test the hypothesis that neighbouring alpha cell paracrine contributions from alpha cell glucagon activate beta cell cAMP, islets from beta cell-specific Ucn3-Cre × lsl-CAMPER mice were dissociated and co-transduced with an adenovirus delivering the red fluorescent Ca^2+^ sensor jRGECO1a to allow the simultaneous tracking of Ca^2+^ and cAMP (Fig. 7a). SST-14 inhibited GIP-induced cAMP and glucose-induced Ca^2+^ in beta cells (Fig. 7b, c), and this response was blocked by the SSTR3 antagonist as expected (Fig 7d, e). However, SSTR2 antagonism no longer increased Ca^2+^ or cAMP (Fig. 7f–o) and ESM Video 7).

### Blocking SST inhibition of alpha cells potentiates insulin release by glucagon and beta cell GLP1R

**Fig. 8:**
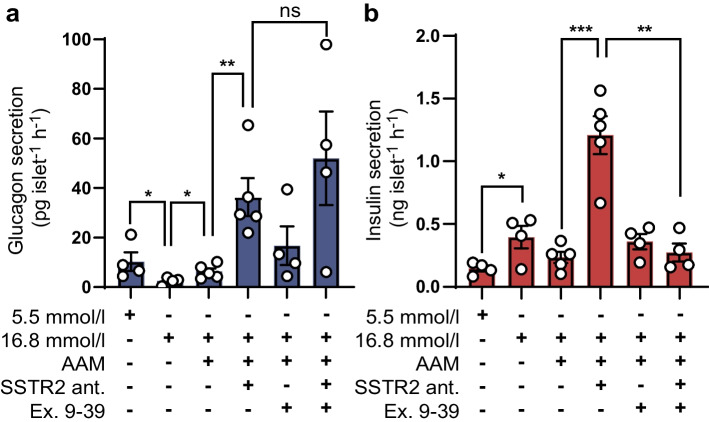
Blocking SST inhibition of alpha cells potentiates insulin release by glucagon via beta cell GLP1R. Insulin and glucagon secretion was acquired from a pooled islet population from 12 mice. (**a**) Glucagon secretion was significantly decreased by high glucose alone but significantly rose when amino acids were added to the high-glucose solution. Glucagon secretion was significantly higher in the presence of 500 nmol/l SSTR2 antagonist. There was no significant difference in glucagon secretion with the addition of the GLP1R antagonist exendin 9-39 (500 nmol/l). (**b**) Insulin secretion was significantly increased by high (16.8 mmol/l) glucose solution. Insulin secretion was significantly higher in the presence of 500 nmol/l SSTR2 antagonist. Addition of exendin 9-39 resulted in a significant decrease in insulin secretion in the presence of the SSTR2 antagonist. All comparisons between groups were performed with the Student’s *t* test between samples; *p<0.05, **p<0.01, ***p<0.001. ant., antagonist; Ex9-39, exendin 9-39; ns, not significant

To validate our imaging results with hormone secretion, we conducted static secretion experiments for glucagon and insulin from the same islets. Stimulation with high glucose alone promoted insulin secretion (Fig. 8b) and caused a significant reduction in glucagon release (Fig. 8a), in line with prior observations [29]. Amino acid addition resulted in a modest but statistically significant increase in glucagon release compared with high glucose alone (Fig. 8a) but did not affect insulin secretion. However, simultaneously blocking alpha cell SSTR2 caused a robust increase in glucagon and insulin secretion (Fig. 8a, b). Co-application of the GLP1R antagonist exendin9-39 abolished insulin secretion while glucagon release remained elevated, demonstrating that SSTR2-controlled glucagon is driving this nutrient-stimulated secretion of insulin.

## Discussion

Our understanding of the alpha cells of the pancreatic islet has expanded from their original textbook description as largely counterregulatory cells responding to hypoglycaemia [2] to encompass a cooperative relationship with beta cells whereby glucagon potentiates nutrient-stimulated insulin secretion [9, 42]. How delta cell feedback engages with this cooperative alpha cell–beta cell relationship to modulate insulin secretion during the postprandial phase has remained unclear. We sought to understand how SST-mediated attenuation of cAMP and Ca^2+^ shapes alpha and beta cell behaviour and paracrine interactions. To accomplish this, we validated islet cell-specific transcriptomes by visualising the SSTR3 protein selectively on beta cell cilia despite robust *Sstr3* mRNA expression in all three islet cell types. Whether alpha cell SSTR3 protein is rapidly targeted for degradation (e.g. by the proteasome via ubiquitin ligases as is the case for p53 or cyclins [43, 44] or via a different mechanism) would be the subject of a future study. We confirmed expression of *Sstr2* mRNA and SSTR2 protein on alpha cells, in line with our own and other groups’ prior observations [16–19].

### SSTR2 is a more potent inhibitor of cell activity than SSTR3

We assessed the relative ability of SST to inhibit Ca^2+^ and cAMP in alpha and beta cells. We expressed genetically encoded sensors for cAMP and Ca^2+^ in both alpha cells and beta cells simultaneously [7, 29]. This revealed that SST inhibited Ca^2^⁺ more strongly in alpha cells than in beta cells. The muted ability of SST to inhibit beta cell Ca^2^⁺ response likely reflects two factors. First, once robustly activated by nutrient stimulation, gap junction connections between beta cells of the intact islet may diminish the effectiveness of the inhibitory effects of SST [7, 45, 46]. This was affirmed by the observation that dissociation of islets made beta cells more sensitive to inhibition by SST. Second, SSTR3 on the beta cell is restricted to the cilia, limiting its’ localised compartment of action with regards to the regulation of whole-cell Ca^2^⁺ [47, 48]. In contrast, cAMP increases driven by GIP were inhibited at a similar magnitude by SST in alpha cells and beta cells. It follows that, in contrast to its effect on Ca^2^⁺, the ciliary localisation of SSTR3 does not restrict whole-cell cAMP decreases in beta cells. This agrees with recent results that documented the ability for ciliary SSTR3 to modulate whole-cell cAMP after SST addition [38]. Additionally, ciliary GPCRs can engage distinct downstream pathways including transcriptional programs compared with membrane-localised receptors such as SSTR2 [38, 49], demonstrating that signalling cascades initiated at the primary cilia can affect change distally in the cell. How cilia-specific activation of Gαi-coupled SSTR3 is able to effectively inhibit cAMP generated in response to GIP or even forskolin, which each presumably activate adenylyl cyclases across the cell, is remarkable and calls for future study.

### SST inhibition of prandial insulin release requires alpha cell-mediated paracrine feedback

Application of SSTR2 and SSTR3 antagonists selectively reversed SST-induced inhibition of alpha cell and beta cell signalling, respectively. However, the SSTR2 antagonist also increased beta cell cAMP within intact islets, which is inconsistent with the lack of SSTR2 on beta cells. The increase in beta cell cAMP resulting from SSTR2 antagonism was abolished by physically separating beta cells from alpha cells by dissociation, indicating that this increase is likely the result of an alpha cell-derived paracrine factor [9, 11]. Indeed, static secretion experiments using intact islets confirmed that delta cell inhibition of alpha cells via SSTR2 controlled downstream beta cell insulin release. Islets isolated from whole-body SSTR2 null mice exhibited no glucose-stimulated insulin secretion impairment but were stimulated in the absence of amino acids that would promote glucagon release [50]. Collectively, our data demonstrate that delta cell feedback under glucose and amino acid co-stimulation restricts insulin release in large part indirectly via alpha cell SSTR2. The presence of an SSTR2 antagonist allows the elevation of alpha cell cAMP, leading to an increase in glucagon release. Local glucagon subsequently activates cAMP in beta cells via GLP1R [42], which we validated by applying the GLP1R antagonist exendin 9-39 in our secretion experiments. Lack of delta cell inhibition of this alpha to beta paracrine potentiation of insulin secretion likely at least partially explains the excessive nutrient-stimulated insulin secretion observed following the deletion of delta cells or removal of SST [31, 51, 52].

### Effects upstream and downstream of SSTR signalling

Our focus was to quantify the relative importance of the inhibition of cAMP and Ca^2+^ second messengers in response to SSTR activation on alpha cells and beta cells by a known concentration of exogenous SST. In so doing, we bypassed endogenous SST release from delta cells in our imaging experiments, although our secretion experiments using only SSTR2 antagonists validated the involvement of endogenous SST. Delta cell activation is influenced by multiple factors, including direct nutrient stimulation, paracrine stimulation by factors such as UCN3, and the influence of Cx36 gap junctions between beta and delta cells that occur at a significantly lower density compared with Cx36 gap junctions between beta cells [7]. Approximately half of all delta cells share few or no Cx36 gap junctions with beta cells and depolarise independently below the beta cell glucose threshold [7]. These delta cells are likely responsible for SST secretion under resting or counterregulatory conditions. In contrast, delta cells with a higher density of gap junctions are restrained until depolarisation by their beta cell neighbours, synchronising their calcium activity with the beta cell pool in the same islet under glucose stimulation [7]. Beta cell loss in type 1 diabetes effectively releases this normally restrained subset of delta cells from the electrochemical inertia imposed by adjacent beta cells. The resulting excess SST suppresses alpha cell activation, contributing to defective counterregulatory glucagon secretion [46].

We investigated the downstream effects of SSTR activation on cAMP and Ca^2^⁺ as if they represent parallel and independent second messenger pathways. This is an oversimplification. The generation of cAMP at local domains interacts directly with L-type calcium channels and gap junctions to modulate excitability and the ensuing calcium dynamics [53, 54]. And of course, Ca^2^⁺ and cAMP are both required for the full process of vesicle recruitment, docking and fusion during exocytosis [55]. This may explain the paradoxical observation that glucose and amino acid co-stimulation of beta cells results in robust Ca^2+^ (Figs 2, 3, 5) without a clear increase in insulin secretion (Fig. 8b). Under these circumstances, the effect of Ca^2+^ on secretion is limited by the number of available secretory granules available at the plasma membrane, rendering the additional calcium stimulated by amino acids superfluous in the absence of new granules at the membrane. SSTR2 antagonism remedies this by promoting glucagon release, which activates beta cell cAMP via GLP1R, which facilitates recruitment of additional secretory granules towards the membrane [56, 57]. This explains why the combination of glucose (to activate beta cell calcium), amino acids (to activate alpha cell calcium) and SSTR2 inhibition (to activate alpha cell cAMP) leads to glucagon secretion, which activates beta cell cAMP and robustly potentiates insulin secretion.

### Study limitations

To ensure robust and homogenous inhibition by SST into the islet interstitial space of isolated islets that lack circulation, we applied SST at approximately tenfold over the IC50 for both SSTR2 and SSTR3 [58]. Antagonists were applied at five times higher molar ratio as in prior studies [31]. While the actions of SSTR2 and SSTR3 antagonists largely conformed to their receptor expression patterns, the transient effect of SSTR3 antagonist treatment on alpha cell Ca^2+^ is unexplained. While a lower-affinity interaction with alpha cell SSTR2 could explain this, we did not observe a similar effect of SSTR3 antagonist treatment on alpha cell cAMP. The use of alpha cell- and beta cell-specific SSTR2 and SSTR3 knockout islets is not feasible for lack of floxed *Sstr2* and *Sstr3* alleles. Global knockout mice for both SSTR2 [50] and SSTR3 [59] are potentially confounded by off-target and possible compensation from any of the other four SSTRs. Future studies should replicate the approaches here in human islets. Previous results examining electrical activity in human alpha and beta cells identified SSTR2 as the functionally dominant receptor [60], which aligns well with our findings in mouse islets. However, the expression profile of SSTRs in human islets includes additional receptors not expressed in mouse islets [61, 62]. As such, disentangling the individual contributions from the human islet SSTR network on the inhibition of Ca^2+^ and cAMP in alpha and beta cells would be a substantial, separate and important undertaking.

### Conclusions

Through examination of the effect of SST on the inhibition of cAMP and Ca^2+^ in alpha cells and beta cells of mouse islets, we have quantified how alpha and beta cell behaviours are attenuated. We demonstrate that SST exerts its strongest receptor-mediated inhibitory effect on insulin secretion in response to nutrient-stimulated postprandial conditions indirectly, by suppressing glucagon-mediated paracrine potentiation of insulin secretion. Importantly, this is distinct from our demonstration that the non-fasting setpoint for glucose in-between meals is determined via a direct and modest feedback inhibition by delta cells that is independent of alpha cells [8, 31]. Taken together, our findings clarify the complex direct and paracrine relationships by which SST affects islet hormone secretion.

## Supplementary Information

Below is the link to the electronic supplementary material.

ESM (PDF 3263 KB)

ESM Video 1 (MP4 21320 KB)

ESM Video 3 (MP4 2214 KB)

ESM Video 4 (MP4 537 KB)

ESM Video 7 (MP4 6091 KB)

## Data Availability

All raw data and analyses represented in this manuscript are available to download indefinitely from the date of publication at https://github.com/Huising-Lab/Hart-et-al.-Diabetologia-2026

## Abbreviations

AAM: Amino acid mixture
FRET: Forster resonance energy transfer
GIP: Gastric inhibitory polypeptide
GLP1R: Glucagon-like peptide-1 receptor
GPCR: G-protein-coupled receptor
ROI: Regions of interest
SST: Somatostatin
SSTR: SST receptor

## Code availability

All code used for analyses and data processing are available on GitHub (https://github.com/Huising-Lab/Hart-et-al.-Diabetologia-2026).
