# Supplementary material for: Somatostatin receptors shape insulin and glucagon output within the pancreatic islet in mice through direct and paracrine effects
Source: Diabetologia. 2026 Jun 20;69(9):2569–84. doi: 10.1007/s00125-026-06769-4 (PMC13423930; doi:10.1007/s00125-026-06769-4)
Supplement: Supplementary file 4 — ESM (PDF 3263 KB) [file 125_2026_6769_MOESM1_ESM.pdf]

Electronic Supplemental Table 1							
**Antibody**	**Species**	**Concentration**	**Company**	**Catalog #**	**RRID**	**Validation**	**Buffer**
polyclonal anti-insulin	Guinea Pig	1:500	Dako	A0564	AB_10013624	PMIDs:38195859,40956879,27408771.	Donkey Block
monoclonal anti-insulin	Rat	1:500	R&D Systems Catalog	MAB1417	AB_2126533	PMIDs:38195859.	Donkey Block
Polyclonal anti-glucagon	Guinea Pig	1:1000	Progen	16032	N/A	PMIDs:38195859.	Donkey Block
Polyclonal anti Somatostatin	Sheep	1:1000	American Research Products	13-2366	AB_1542966	PMIDs:38195859.	Donkey Block
Polyclonal anti somatostatin receptor 2	Rabbit	1:1000	Alomone	ASR-006	AB_2040208	Aligns with mRNA results and cell specific behavior	Donkey Block
Polyclonal anti somatostatin receptor 3	Rabbit	1:1000	ThermoFisher	PA3-207	AB_10981488	Aligns with mRNA results and ciliary localization	Donkey Block
Polyclonal anti-GFP	Goat	1:1000	Rockland	600-101-215	AB_218182	PMIDs:38195859,40956879,27408771.	Donkey Block
Cy3-AffiniPure F(ab')2 Fragment Anti-Guinea Pig IgG	Donkey	1:600	Jackson Immunoresearch	706-166-148	AB_2340461	Complete colocalization with target species, secondary only control	Donkey Block
647-AffiniPure F(ab')2 Fragment Anti-Guinea Pig IgG	Donkey	1:600	Jackson Immunoresearch	706-606-148	AB_2340477	Complete colocalization with target species, secondary only control	Donkey Block
Cy3-AffiniPure F(ab')2 Fragment Anti-Rat IgG	Donkey	1:600	Jackson Immunoresearch	712-166-153	AB_2340669	Complete colocalization with target species, secondary only control	Donkey Block
647-AffiniPure F(ab')2 Fragment Anti-Rat IgG	Donkey	1:600	Jackson Immunoresearch	712-606-153	AB_2340696	Complete colocalization with target species, secondary only control	Donkey Block
488-AffiniPure F(ab')2 Fragment Anti-Goat IgG	Donkey	1:600	Jackson Immunoresearch	705-546-147	AB_2340430	Complete colocalization with target species, secondary only control	Donkey Block
647-AffiniPure F(ab')2 Fragment Anti-Sheep IgG	Donkey	1:600	Jackson Immunoresearch	711-606-152	AB_2340625	Complete colocalization with target species, secondary only control	Donkey Block
Cy3-AffiniPure F(ab')2 Fragment Anti-Rabbit IgG	Donkey	1:600	Jackson Immunoresearch	711-166-152	AB_2313568	Complete colocalization with target species, secondary only control	Donkey Block
647-AffiniPure Anti-Rabbit IgG	Donkey	1:600	Jackson Immunoresearch	711-605-152	AB_2492288	Complete colocalization with target species, secondary only control	Donkey Block

Donkey Block Formula

Potassium Phosphate Buffered Saline (KPBS) supplemented with: 0.4% Triton-X 100 (Vol/Vol), 0.2% Donkey Serum (Vol/Vol)

ESM Figure 1

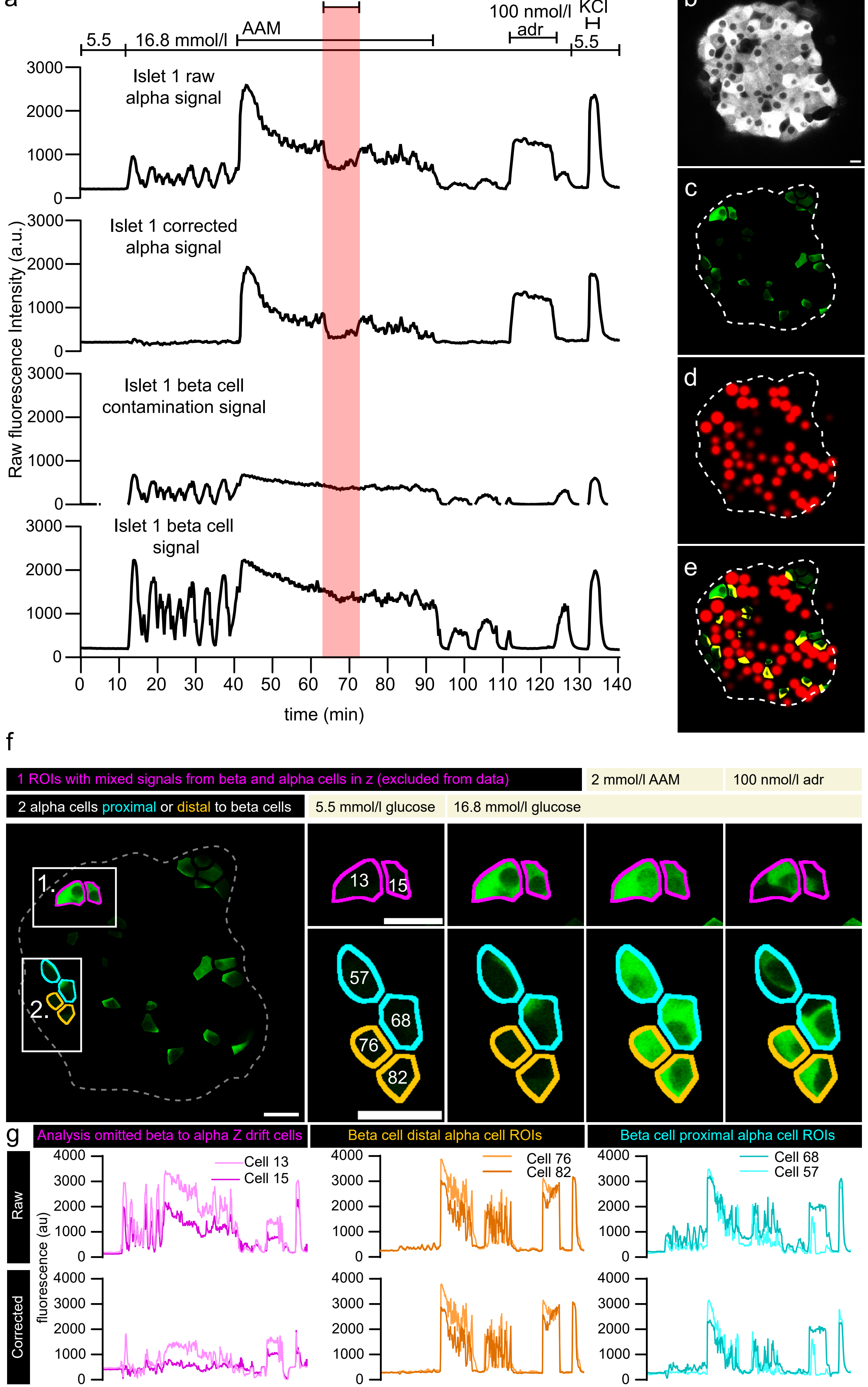
a
100 nmol/l SST
30 mmol/l KCl
100 nmol/l adr
5.5
16.8 mmol/l
AAM
5.5
Islet 1 raw alpha signal
Islet 1 corrected alpha signal
Islet 1 beta cell contamination signal
Islet 1 beta cell signal
Raw fluorescence Intensity (a.u.)
time (min)
b
c
d
e
f
1 ROIs with mixed signals from beta and alpha cells in z (excluded from data)
2 mmol/l AAM
100 nmol/l adr
2 alpha cells proximal or distal to beta cells
5.5 mmol/l glucose
16.8 mmol/l glucose
1.
2.
13
15
57
68
76
82
g
Analysis omitted beta to alpha Z drift cells
Beta cell distal alpha cell ROIs
Beta cell proximal alpha cell ROIs
Raw
Corrected
fluorescence (au)
Cell 13
Cell 15
Cell 76
Cell 82
Cell 68
Cell 57

Electronic Supplemental Figure 1: Correction of β cell contamination of neighbouring α cells in Ca^{2+} imaging experiments. Sequential graphs highlighting the average raw signal of α cell ROIs in a representative islet are presented (a). The corrected signal and contamination signal demonstrate the portions of the raw signal which account for the true α cell signal (corrected) and β cell contamination of the ROIs (contamination signal) (a). The averaged raw signal from the β cell ROIs of the same islet is presented as a comparison for the removed contamination signal from the α cells (a). A raw still of the islet presented in the graphs (a), is shown in just 16.8 mmol/l glucose (b) (scale bar 10μm). Unbiased ROIS of α cells identified by their response to adrenaline are isolated from the same time point as the raw still (c). Neighbouring β cells are identified for assessing their contamination contributions (d). The contamination signal at this time point is presented with regions of high contamination in α cells from neighbouring β cells highlighted in yellow (e). A supplemental movie (Supplemental Movie 1) is provided to view these features over the course of an entire imaging experiment. Three sets of α cell ROIs are presented, a set that represents a pair of omitted cells due to their drift from β cell to α cell in Z during the experiment (box 1, magenta ROIs) and a set of α cells that are proximal (teal) and distal (orange) to β cells (box 2) during the experiment (f) (scale bars 20μm. Plotting of the raw and cleaned α cell intensity data for the highlighted cells (f) illustrates the inability for this approach to process β cell ROIs in the absence of α cells (g) (magenta). The distal (orange) α cells display less β cell contamination than the proximal α cells (teal), which is reflected in the absence or presence of β cell contamination removal from the raw to corrected line graphs (g). adr, adrenaline

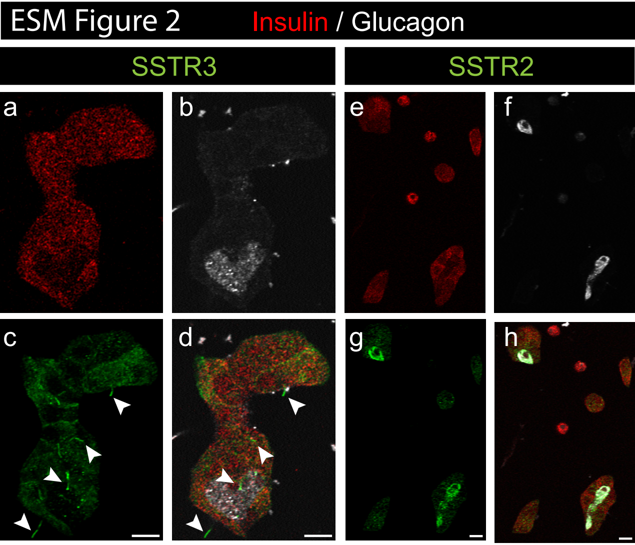

ESM Figure 2
Insulin / Glucagon
SSTR3
SSTR2
a
b
c
d
e
f
g
h

Electronic Supplemental Figure 2: Dissociation of primary mouse islets does not alter SSTR3 ciliary or SSTR2 α cell localization. Dissociated primary mouse islets retain their primary cilia (a-d) (scale bar 10μm). Dissociated primary mouse islets retain the α cell specificity for SSTR2 (e-h) (scale bar 10μm).

Electronic Supplementary Material – Videos

ESM Video 1 – Video of an islet expressing GCaMP6s in all islet cells. This video illustrates how the alpha cell ROI cleanup method is employed. The left pane is a grayscale image of the acquired multi-image movie to illustrate live dynamics. The right pane displays all alpha cell ROIs isolated in green. The nearest neighbor beta cells are displayed as red, with their signal propagation and intensity shown as red circles with light diffusing away. The identified alpha cell signal contamination from neighboring beta cells is highlighted in yellow. Accompanies ESM Figure 1.

ESM Video 2 – Video of an islet expressing GCaMP6s in all cells. Accompanying audio is compiled from the average GCaMP6s response with beta cells being represented by the lower pitch and alpha cells by the higher pitch. Islet is exposed to 5.5mM glucose for 10 minutes before treatment with 16.8mM glucose for 105 minutes, with 2 mmol/l of L-glutamine, L-alanine and L-arginine each (AAM) added to the high glucose solution at the 40-minute mark for 45 minutes. At the 60-minute mark. 100 nmol/l SST is applied for 10 minutes to inhibit alpha and beta cells. After 110 minutes, 100nmol/l adrenaline (epinephrine) is applied for 10 minutes to inhibit beta and activate alpha cells. After adrenaline (epinephrine) pulse, islet is perfused with 5.5mM glucose to quiet alpha and beta cells. 30mM KCl is pulsed at the 130 minute-mark to confirm cell viability. Accompanies Figure 2.

ESM Video 3 – Video of dissociated islet cells expressing GCaMP6s in all cells. Accompanying audio is compiled from the average GCaMP6s response with beta cells being represented by the lower pitch and alpha cells by the higher pitch. Islet is exposed to 5.5mM glucose for 10 minutes before treatment with 16.8mM glucose for 105 minutes, with 2 mmol/l of L-glutamine, L-alanine and L-arginine each (AAM) added to the high glucose solution at the 40-minute mark for 45 minutes. At the 60-minute mark. 100 nmol/l SST is applied for 10 minutes to inhibit alpha and beta cells. After 110 minutes, 100nmol/l adrenaline (epinephrine) is applied for 10 minutes to inhibit beta and activate alpha cells. After adrenaline (epinephrine) pulse, islet is perfused with 5.5mM glucose to quiet alpha and beta cells. 30mM KCl is pulsed at the 130 minute-mark to confirm cell viability. Accompanies Figure 3.

ESM Video 4 – Video representing an islet expressing the cAMP sensor H74 in all islet cells (cCAMPER mouse). To improve visibility of cell types alpha cell ROIs are pseudo colored blue, with beta cells pseudo colored red. White hues represent low cAMP and bright red or blue hues represent high cAMP. Islet is maintained at 5.5 mM glucose for 40 minutes. To increase cAMP in both alpha and beta cells, 100 nmol/l GIP is applied from the 5-minute to 40-minute time point. 100 nmol/l of SST is applied from the 15 to 25-minute time point, with washout following for 10 minutes. 100 nmol/l of adrenaline

(epinephrine) is applied from the 35 to 40-minute time point to activate alpha and inhibit beta cells. Accompanies Figure 4

ESM Video 5 – Video of an islet expressing GCaMP6s in all cells. Accompanying audio is compiled from the average GCaMP6s response with beta cells being represented by the lower pitch and alpha cells by the higher pitch. Islet is exposed to 5.5mM glucose for 10 minutes before treatment with 16.8mM glucose for 150 minutes, with 2 mmol/l of L-glutamine, L-alanine and L-arginine each (AAM) added at the 30-minute mark on top of the high glucose solution for 100 minutes. At the 50-minute mark, 100 nmol/l SST is applied for 65 minutes to inhibit alpha and beta cells and establish an “inhibitory baseline”. From 65 to 75 minutes, 500 nmol/l SSTR2 antagonist and from 95 to 105 500 nmol/l SSTR3 antagonist is applied against the inhibitory baseline. At the 150-minute mark, 100 nmol/l adrenaline (epinephrine) is applied for 10 minutes to inhibit beta and activate alpha cells. Accompanies Figure 5.

ESM Video 6 – Video representing an islet expressing the cAMP sensor H74 in all islet cells (cCAMPER mouse). To improve visibility of cell types alpha cell ROIs are pseudo colored blue, with beta cells pseudo colored red. White hues represent low cAMP and bright red or blue hues represent high cAMP. Islet is maintained at 5.5 mM glucose for 110 minutes. To increase cAMP in both alpha and beta cells, 100 nmol/l GIP is applied from the 5-minute to 110-minute time point. 100 nmol/l of SST is applied from the 20 to 110-minute time point to establish an “inhibitory baseline”. From 30 to 45 minutes 500 nmol/l SSTR2 antagonist and from 65 to 80 minutes SSTR3 antagonist is applied against the inhibitory baseline. 100 nmol/l of adrenaline (epinephrine) is applied from the 100 to 110-minute time point to activate alpha and inhibit beta cells. Accompanies Figure 6

ESM Video 7 – Video of dissociated *Ucn3-cre x lsl-cAMPER* islet cells expressing the cAMP sensor H74 exclusively beta cells on the left. Dissociated cells are transduced with the red calcium indicator jRGECO1a, with visualization presented on the right. Cells are exposed to 5.5mM glucose for 10 minutes before treatment with 11mM glucose for 100 minutes at the 10-minute mark. At the 30-minute mark, 100 nmol/l GIP is applied for 70 minutes. 100 nmol/l SST is applied for 50 minutes to inhibit alpha and beta cells, establishing an inhibitory baseline for both cAMP and calcium recordings. An SSTR3 antagonist is applied from 50 to 60 minutes and an SSTR2 antagonist is applied from 70-80 minutes against the inhibitory baseline. After 110 minutes, cells are returned to 5.5mM glucose, with 30mM KCl and 1uM forskolin applied at the 120- and 125-minute marks respectively to confirm cell viability. Accompanies Figure 7.
